# Denosumab Regulates Gut Microbiota Composition and Cytokines in Dinitrobenzene Sulfonic Acid (DNBS)-Experimental Colitis

**DOI:** 10.3389/fmicb.2020.01405

**Published:** 2020-06-25

**Authors:** Azin Khafipour, Nour Eissa, Peris M. Munyaka, Mohammad F. Rabbi, Kunal Kapoor, Laetitia Kermarrec, Ehsan Khafipour, Charles N. Bernstein, Jean-Eric Ghia

**Affiliations:** ^1^Department of Immunology, University of Manitoba, Winnipeg, MB, Canada; ^2^Children’s Hospital Research Institute of Manitoba, University of Manitoba, Winnipeg, MB, Canada; ^3^Section of Gastroenterology, Department of Internal Medicine, Rady Faculty of Health Sciences, University of Manitoba, Winnipeg, MB, Canada; ^4^University of Manitoba IBD Clinical and Research Centre, University of Manitoba, Winnipeg, MB, Canada; ^5^Department of Animal Science, University of Manitoba, Winnipeg, MB, Canada

**Keywords:** IBD, immunotherapy, immune responses, gut microbes, TNF

## Abstract

The pro-inflammatory mediator receptor activator of nuclear factor-kappa B ligand (RANKL) plays a significant role in the development of rheumatoid arthritis; however, its role in inflammatory bowel disease is unknown. Genome-wide association meta-analysis for Crohn’s disease (CD) identified a variant near the TNFSF11 gene that encodes RANKL and CD risk allele increased expression of RANKL in specific cell lines. This study aims to elucidate if the RANKL inhibitor denosumab can reduce the severity of experimental colitis and modify the gut microbiota composition using murine dinitrobenzenesulfonic acid (DNBS)-experimental model of colitis mimicking CD. In colitic conditions, denosumab treatment significantly decreased the pro-inflammatory cytokines IL-6, IL-1β, and TNF-α within the colonic mucosa. Moreover, colitis was accompanied by disruption of gut microbiota, and preventative treatment with denosumab modulated this disruption. Denosumab treatment also modified the alpha- and beta diversity of colonic mucosa and fecal microbiota. These results provide a rationale for considering denosumab as a future potential therapy in CD; however, more detailed experimental and clinical studies are warranted.

## Introduction

Inflammatory bowel disease (IBD) consists of two major types of intestinal disorders, Crohn’s disease (CD) and ulcerative colitis (UC) that are characterized by chronic inflammation and ulceration in different segments of the gastrointestinal tract (Kelsen and Sullivan, 2017; Nemati and Teimourian, 2017). The prevalence of IBD has been increasing in industrialized countries; therefore, increasing medical costs mostly secondary to expensive biological therapies (Olivera et al., 2019). For instance, the direct medical costs of IBD in Canada alone exceeded $CAD 1.28 billion per annum (Kuenzig et al., 2019). While no curative therapy is available for IBD, the current therapies aim to maintain remission via modulation of the immune system (e.g., corticosteroids), suppression of inflammatory cytokines (e.g., TNF-α blockers), and/or regulation of the gut microbiome (e.g., intestinal microbiota transplantation). However, these therapies have potential side effects (Sartor, 2004; De Souza and Fiocchi, 2016); and as such there is demand for safe and cost-effective therapeutic strategies for IBD. Despite the uncertainty in the etiology of IBD, the success of modulating immune responses and the gut microbiome suggests that both intestinal immune dysregulation and gut dysbiosis are involved in IBD pathogenesis (Kelsen and Sullivan, 2017; Lopetuso et al., 2017; Nemati and Teimourian, 2017; Rodriguez de Santiago et al., 2017; Rogler, 2017).

The healthy balance of the human gut microbiome is essential for maintaining the host equilibrium state (Qin et al., 2010; D’argenio and Salvatore, 2015). Recently, the etiology of the aberrant immune response in the context of gut microbial dysbiosis has been demonstrated (Degruttola et al., 2016). Dysbiosis mostly refers to an imbalance in robustness or the resilience of the quasi-stable state of the microbes residing within the gut (Backhed et al., 2012; Flores et al., 2014). Others and we have shown that acute dextran sulfate sodium (DSS)-induced model of UC is associated with alterations in the composition and reduced functionality of murine gut microbiota (Brinkman et al., 2013; Munyaka et al., 2016a). In DSS-induced colitis, colonic microbiota changes have correlated with several alterations of the immune system (Brinkman et al., 2013; Håkansson et al., 2015). The intrarectal administration of trinitrobenzensulfonic acid (TNBS), an experimental model of CD, also results in colon and feces dysbiosis (He et al., 2016).

Genome-wide association studies (GWAS) have reported 163 genes and genetic loci that can contribute to IBD pathogenesis (Abreu, 2013; Loddo and Romano, 2015; Liu and Stappenbeck, 2016) of which approximately 30% are shared between CD and UC patients. An example is a variant near the gene TNFSF11 that encodes for receptor activator of nuclear factor kappa-B ligand (RANKL) of which the expression is increased in CD patients (Franchimont et al., 2004; Krela-Kazmierczak et al., 2016) but not in UC patient when compared with control (Stanisławowski et al., 2014). While RANKL has been shown to play a major role in the development of osteoporosis by promoting excessive osteoclastogenesis, its role in the development of CD is not clear. Due to the role played by the RANK/RANKL axis in the pro-inflammatory pathway of the immune system, it is expected that this pathway could also contribute to the regulation of CD (Ke et al., 2019).

Denosumab, a humanized monoclonal antibody against RANKL (Fouque-Aubert and Chapurlat, 2008) has been developed as an effective treatment for postmenopausal osteoporosis (PMO) with high fracture risk (Dempster et al., 2012). Through binding to RANKL, denosumab can compensate for the lack of osteoprotegerin (OPG) concentration in the bone remodeling process and reduce bone resorption by reducing the activation of osteoclasts (Miller, 2011; Dempster et al., 2012). Denosumab is considered as an appropriate first-line pharmacologic option for PMO management due to its efficacy and safety (Anastasilakis et al., 2009; Miller, 2011; Josse et al., 2013). We hypothesized that administration of denosumab attenuates the adverse effects of dinitrobenzosulfonic acid (DNBS)-induced colitis via regulation of immune activation and gut microbiota composition. Results demonstrated that the intraperitoneal administration of the RANKL inhibitor alters the composition and functionality of colonic and fecal microbial communities and downregulates the inflammatory response. Therefore, RANKL inhibitor could potentially serve as a therapeutic for UC treatment.

## Materials and Methods

### Animals and Experimental Design

**FIGURE 1:**
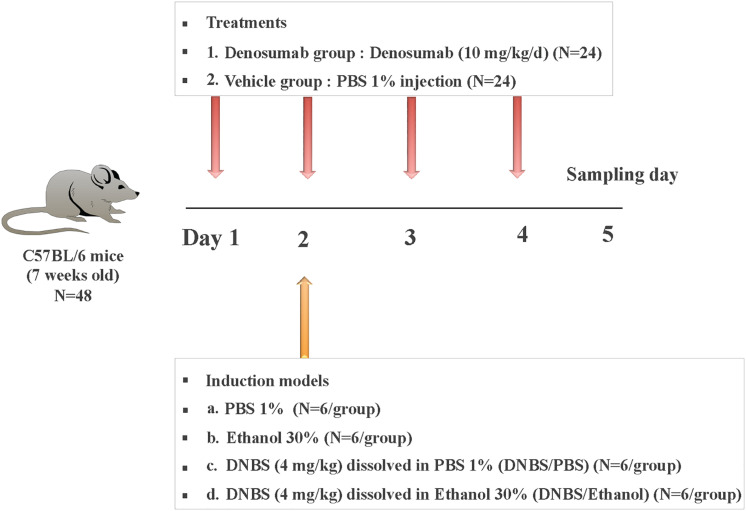
Experimental design. Mice were treated with daily injection (i.p.) of PBS 1% (vehicle; *n* = 24) or denosumab (*n* = 24) at 10 mg/kg/d for a total of 4 days. On day 2 of the experiment, mice were divided into four subgroups (*n* = 6/subgroup) and subjected to different colitis induction models: (a) PBS 1%, (b) Ethanol 30%, (c) DNBS (4 mg/kg) dissolved in PBS 1% (DNBS/PBS), and (d) DNBS (4 mg/kg) dissolved in Ethanol 30% (DNBS/Ethanol). On three days later on day 5, all mice were sacrificed, and colon tissue/mucosa and feces samples were collected.

Forty-eight, 7-week-old male C57Bl/6 mice were maintained under co-housed pathogen-free conditions in the animal care facility at the Faculty of Health Sciences, University of Manitoba, Winnipeg, MB, Canada. Mice received daily intraperitoneal (i.p.) injections of phosphate-buffered saline (PBS) 1% (vehicle) or denosumab at 10 mg/kg/d (Hofbauer et al., 2009; Kostenuik et al., 2009) for 4 days. On day two of the experiment, mice were divided into subgroups (*n* = 6/subgroup) and subjected to different treatments: (a) 1% PBS, (b) 30% Ethanol, (c) DNBS (4 mg/kg) dissolved in 1% PBS (DNBS/PBS), and (d) DNBS (4 mg/kg) dissolved in 30% Ethanol (DNBS/Ethanol) (Figure 1). Injections were done intrarectally using a PE-90 tubing (10 cm long; ClayAdam, Parisppany, NJ, United States) inserted 3.5 cm into their colons and attached to a tuberculin syringe (BD, Mississauga, ON, Canada). All mice received a similar standard chow diet. The experimental protocol (15–010) was approved by the University of Manitoba Animal Ethics Committee and conducted under the guidelines of the Canadian Council on Animal Care (Canadian Council on Animal Care [CCAC], 2009).

### Disease Activity Index and Macroscopic Score

Disease activity index (DAI), a composite index taking into consideration the percentage of weight loss, stool consistency, and fecal blood scores, was assessed from day 0 to day 4. The DAI scoring system was defined as follows: weight: 0, no loss; 1, 5–10%; 2, 10–15%; 3, 15–20%; and 4, >20%; stool: 0, normal; 2, loose stool; and 4, diarrhea; and bleeding: 0, no blood; 2, presence of blood; and 4, gross blood. The presence of blood in the stool was assessed using the Hemoccult II test (Beckman Coulter, Oakville, ON, Canada).

On day 3 post-induction of colitis day, the colon was opened longitudinally, and macroscopic damages were assessed immediately using a previously established scoring system (Cooper et al., 1993; Khan et al., 2002). Macroscopic scores were evaluated based on four parameters, including rectal bleeding, rectal prolapse, diarrhea, and colonic bleeding. Histology analysis was assessed using fixed colonic segments that were paraffin (Sigma, Mississauga, ON, Canada) –embedded and then stained (10 μm sections) using hematoxylin-eosin (H&E) (Sigma). Architectural modifications, goblet cell depletion, edema/ulceration and degree of inflammatory cells infiltrate were considered as evaluating the inflammatory response (Ghia et al., 2009).

### Serum- CRP and Colonic MPO and Cytokine Assessment

Under isoflurane (Abbott, Mississauga, ON, Canada) anesthesia, blood was collected through intracardiac puncture and serum C-reactive protein (CRP) was assessed. Colonic inflammatory cytokines were assessed after colon samples homogenization in Tris-HCl buffer containing protease inhibitors (Sigma). The supernatant was frozen at −80 °C until assay. Serum CRP, colonic myeloperoxidase activity (MPO) (Hycult Biotech, PA, United States) level and cytokine concentrations [interleukin (IL)-6, IL-1β, tumor necrosis factor (TNF)-α] were quantified using enzyme-linked immunosorbent assays (ELISA) using commercial kits (R&D Systems, Inc., Minneapolis, MN, United States) according to the manufacturer instructions (Eissa et al., 2016).

### Quantitative Reverse Transcription Polymerase Chain Reaction (qRT-PCR) for Mucosal Cytokine Evaluation

RNA extraction using TRIzol (Gibco BRL, Life Technologies, NY, United States) was performed using approximately 30–40 mg of colon tissue. Quality and quantity of RNA were determined by measuring the absorbance at 260 and 280 nm spectrophotometrically (NanoDrop ND-1000 UV-Vis, Thermo Fisher Scientific). All samples had an absorption ratio A_260/280_ greater than 1.8. Reverse transcription was performed using SuperScript VILO cDNA Synthesis Master Mix (Invitrogen, Grand Island, NY, United States) in an Eppendorf Thermo cycler at 25 °C for 10 min, followed by 42 °C for 60 min, and 85°C for 5 min according to the manufacturer’s instructions. Samples were stored at −20 °C for qRT-PCR analysis. qRT-PCR reactions were performed in a Roch light Cycler 96 Real-Time System using Power SYBR green master mix (Life Technologies, Burlington, ON, Canada) in a final volume of 20 μL reactions. qRT-PCR conditions were as follows: 95°C for 10 min, followed by 40 cycles at 95°C for 15 s and at 60°C for 60 s. As the reference gene, the TATA Box Binding Protein (tbp) primer (forward ACCGTGAATCTTGGCTGTAAAC, reverse GCAGCAAATCGCTTGGGATTA) (Eissa et al., 2016; Eissa et al., 2017), and *il1b* (forward, GCAACTGTTCCTGAACTCAACT reverse ATCTTTTGGGGTCCGTCAACT), *il6* (forward, TAG TCCTTCCTACCCCAATTTCC reverse TTGGTCCTTAGCC ACTCCTTC) and *tnf*α (forward CCCTCACACTCAGATCAT CTTCT, reverse GCTACGACGTGGGCTACAG) were used, designed from nucleotide sequences identified using NCBI BLAST^1^ (Eissa et al., 2016; Eissa et al., 2017). All qRT-PCRs were run in duplicate; the average standard deviation within duplicates of all samples studied was 0.25 cycles.

### Fecal and Mucosal Microbiota DNA Extraction and Quality Control

Colon mucosa and fecal samples were homogenized at room temperature and their DNA was extracted using ZR Tissue and Insect DNA extraction Kit (Zymo Research Corp., Orange, CA, United States) and ZR fecal DNA extraction kit (Zymo Research Corp.), respectively. Bead-beating step for mechanical lysis of the microbial cells was included in both kits and NanoDrop 2000 spectrophotometer was used (ThermoFisher Scientific, Wilmington, DE, United States). DNA purity was assessed by measuring A_260/280_ while DNA quality was evaluated by agarose gel electrophoresis following PCR amplification of the 16S rRNA gene using universal primers as previously described (Eissa et al., 2017).

### Library Construction and Illumina Sequencing

Library construction and Illumina sequencing were performed as described [39]. Briefly, the V4 region of 16S rRNA gene was targeted for PCR amplification using modified F515/R806 primers (Caporaso et al., 2012), as previously described (Khafipour et al., 2009; Derakhshani et al., 2016a). Briefly, the reverse PCR primer was indexed with 12-base Golay barcodes allowing for multiplexing of samples. The PCR reaction for each sample was performed in duplicate and contained 1.0 μL of pre-normalized DNA (20ng/μL), 1.0 μL of each forward and reverse primers (10 μM), 12 μL high-quality reagents and chemicals (HPLC) grade water (Thermo Fisher Scientific), and 10 μL 5 Prime Hot MasterMix (5 Prime Inc., Gaithersburg, MD, United States). Reactions consisted of an initial denaturing step at 94°C for 3 min followed by 35 amplification cycles at 94°C for 45 sec, 50°C for 60 sec, and 72°C for 90 sec, and an extension step at 72°C for 10 min in an Eppendorf Mastercycler pro (Eppendorf, Hamburg, Germany). PCR products were then purified using ZR-96 DNA Clean-up Kit (ZYMO Research) to remove primers, deoxyribosenucleotides (dNTPs), and reaction components. The V4 library was then generated by pooling 200 ng of each sample and quantified using Picogreen (Invitrogen, Burlington, NY, United States). This was followed by multiple dilution steps using pre-chilled hybridization buffer (HT1; Illumina, San Diego, CA, United States) to bring the pooled amplicons to a final concentration of 5 pM, measured by Qubit2.0 Fluorometer (Life technologies). Finally, 15% of PhiX control library was spiked into the amplicon pool to improve the unbalanced and biased base composition, a known characteristic of low diversity 16S rRNA libraries. Customized sequencing primers for read1 (5′-T ATGGTAATTGTGTGCCAGCMGCCGCGGTAA-3′), read2 (5′- AGTCAGTCAGCCGGACTACHVGGGTWTCTAAT-3′), and index read (5′-ATTAGAWACCCBDGTAGTCCGGCTGACTGA CT-3′; Integrated DNA Technologies, Coralville, IA, United States) were added to the MiSeq Reagent V2 Kit (300-cycle; Illumina). The 150 paired-end sequencing reaction was performed on a MiSeq platform (Illumina) at the Gut Microbiome and Large Animal Biosecurity Laboratories (Department of Animal Science, University of Manitoba, Winnipeg, MB, Canada). The sequencing data are uploaded into the Sequence Read Archive (SRA) of NCBI^2^) and can be accessed through accession numbers SRR1158862–SRR11588957.

### Bioinformatics Analyses

Bioinformatics analyses were performed as described previously (Munyaka et al., 2016a). Briefly, the PANDAseq assembler (Masella et al., 2012) was used to merge overlapping paired-end Illumina fastq files. The output fastq file was then analyzed using downstream computational pipelines in the open-source software package QIIME 1 v.9 (Caporaso et al., 2010). Chimeric reads were filtered using UCHIME (Edgar et al., 2011), and sequences were assigned to Operational Taxonomic Units (OTU) using the QIIME 1 implementation of UCLUST (Edgar, 2010) at 97% pairwise identity threshold using an open reference OTU picking process (Rideout et al., 2014). Taxonomies were assigned to the representative sequence of each OTU using an RDP classifier (Wang et al., 2007) and aligned with the Greengenes (v. 13.5) core reference database (Desantis et al., 2006) using PyNAST algorithms (Caporaso et al., 2010). To compare microbial communities, the phylogenetic tree was built with FastTree 2.1.3. (Price et al., 2010).

### Alpha and Beta Diversity

Within-community diversity (α-diversity) was calculated by different indices of species richness and evenness including Chao1 and Shannon, using the open-source bioinformatics package QIIME 1 (Caporaso et al., 2010) and Phyloseq R package (3.1.0) (Mcmurdie and Holmes, 2013). The p-values were calculated, using the MIXED procedure of SAS (SAS 9.3) using a randomized factorial design where the effects of treatment (vehicle vs. Denosumab), induction model (PBS 1%, Ethanol 30%, DNBS/PBS, DNBS/Ethanol), and their interaction were considered as fixed factors and the effect of mice as a random factor. Even depth of 1,500 and 25,000 sequences per sample were used to calculate the richness and diversity indices for the colon mucosa and feces, respectively. To assess the beta-diversity (β-diversity) differences among bacterial communities from different treatments within each induction model, non-metric multidimensional scaling (nMDS) ordination plots were generated using R software (3.1.1) by employing Bray-Curtis similarity matrices with a conventional cut-off of <0.2 for the stress value (Munyaka et al., 2016b). The resulting minimum stress solution was used to produce the nMDS plots, in which each data point represents one sample. The spatial distance between points in the plot was interpreted as the relative difference in the bacterial community composition; thus, points that were closer were more similar than points that were more distant. To assess the statistical differences in β-diversity of bacterial communities among treatment groups, permutation multivariate analysis of variance (PERMANOVA) (Anderson, 2005) was performed using the above-mentioned statistical model, and p-values were calculated.

### Clustering Analysis

To illustrate the distinct clustering pattern within colonic vehicle and denosumab groups, the relative abundance of the OTUs were binned into genus-level taxonomic groups and filtered to keep the most abundant genera found across all samples (cutoff value of greater than 0.1% of the community) (Derakhshani et al., 2016b). The resulting relative abundance table was normalized to correct for compositionality and also assist heat map-visualization of differentially abundant genera. The dissimilarity of samples were calculated based on Bray–Curtis measures using R “vegan” package (Oksanen et al., 2007) and the resulting matrix was subjected to unsupervised hierarchical clustering using R “dendextend” package (Galili, 2015) and visualized over the heat map of abundance matrix using R “complexheatmap” package (Gu et al., 2016). Genera were also clustered based on their Spearman’s correlation coefficient using R “complexheatmap” package.

### Correlation Coefficients

Associations between bacterial taxa with an abundance ≥0.5% of the community in the colon mucosa, inflammatory markers (IL-1β, IL-6, TNF-α, CRP, MPO) and tight junction proteins (filamentous actin, F-actin; and occluding, OCl) and alpha-diversity indices were explored using non-parametric Spearman’s rank correlation implemented in PAST software (Hammer et al., 2001). For each correlation, correlation coefficient (Spearman’s Rho) and p-value were obtained (Wei and Simko, 2016) and the resulting correlation matrix was visualized in a heatmap format^3^. The correlation coefficient values ranged from -1 to +1 with larger absolute values indicating stronger relationship while positive and negative values indicating the direction of association. Alpha value for the correlation confidence intervals was set up as 0.05.

### Prediction of Functional Metagenomics

The open-source software PICRUSt (v. 1.0.0-dev) was used to predict the functional capacity of microbiome using 16S rRNA gene sequencing data and Greengenes (v. 13.5) reference database (Desantis et al., 2006). To make our open-reference picked OTUs compatible with PICRUSt, all de-novo OTUs were removed and only those that had matching Greengenes identifications were retained. The new OTU table was then used to generate metagenomic data after normalizing the data by copy numbers, and to derive relative Kyoto Encyclopedia of Genes and Genomes (KEGG) pathway abundance (Langille et al., 2013). The KEGG data was analyzed using STAMP (v. 2.1.3) (Parks et al., 2014).

### Other Statistical Analysis

Normality of residuals for α-diversity and inflammatory markers was tested in the SAS UNIVARIATE procedure (SAS 9.3, 2012). Non-normally distributed data were log transformed. Original or transformed data were further analyzed using SAS MIXED procedure with the effects of treatment (vehicle vs. denosumab), induction model (PBS 1%, Ethanol 30%, DNBS/PBS, DNBS/Ethanol), and their interaction as fixed factors and mice as a random factor. Tukey studentized range adjustment was used for all pairwise comparisons among the groups. GraphPad Prism (v. 6, GraphPad Software Inc. La Jolla, CA, United States) was used to plot the inflammatory variables graphs using the multiple comparisons of two-way analysis of variance (ANOVA). Differences were reported as significant when *p* < 0.05 while trends were discussed at 0.05 ≤ *p* < 0.1. For inflammatory markers, when differences among treatments were significant, the magnitude of change was expressed as fold-change.

## Results

### Effect of Denosumab Treatment on the Disease Activity Index

**FIGURE 2:**
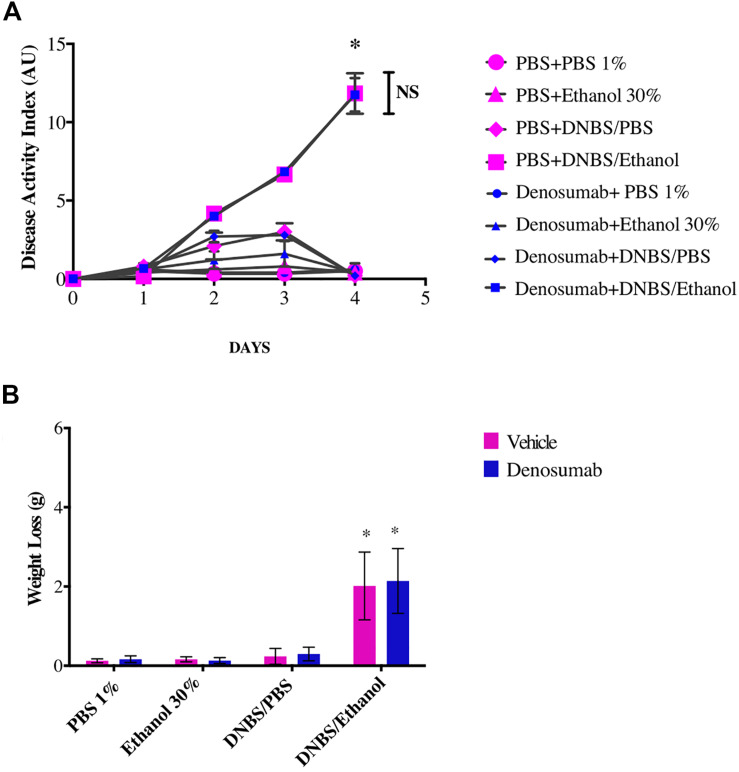
The effects of DNBS/Ethanol-induced colitis and denosumab treatment on **(A)**. Disease activity index, and **(B)**. Weight loss. Vehicle = PBS 1%. Error bars are shown as SEM. *p < 0.001 when compared with other induction models within the denosumab or vehicle group calculated using SAS MIXED procedure and illustrated by Prism using two-way ANOVA. NS: no significant difference between denosumab treatment and DNBS/Ethanol-induced colitis. *n* = 6 mice per group. AU: arbitrary unit.

PBS 1%, Ethanol 30% or DNBS/PBS treatments did not affect the development of colitis across treatments. However, compared to ethanol treatment, DNBS/Ethanol induced a colitis characterized by an increase of the DAI (*p* = 0.02), weight loss (*p* = 0.01), blood presence in the feces and a decrease of stool consistency (Figures 2A,B). This was evident from day 2 to day 4 post-colitis induction (Figures 2A,B), where DAI increased by 34.5-fold (*p* = 0.03). In non-colitic conditions (PBS 1%, Ethanol 30%, DNBS/PBS), and colitic conditions (DNBS/Ethanol), denosumab did not affect any of weight loss (Figure 2B), or stool consistency and presence of blood in the feces (Figure 2A).

### Effect of Denosumab Treatment on the Macroscopic and Histological Scores

**FIGURE 3:**
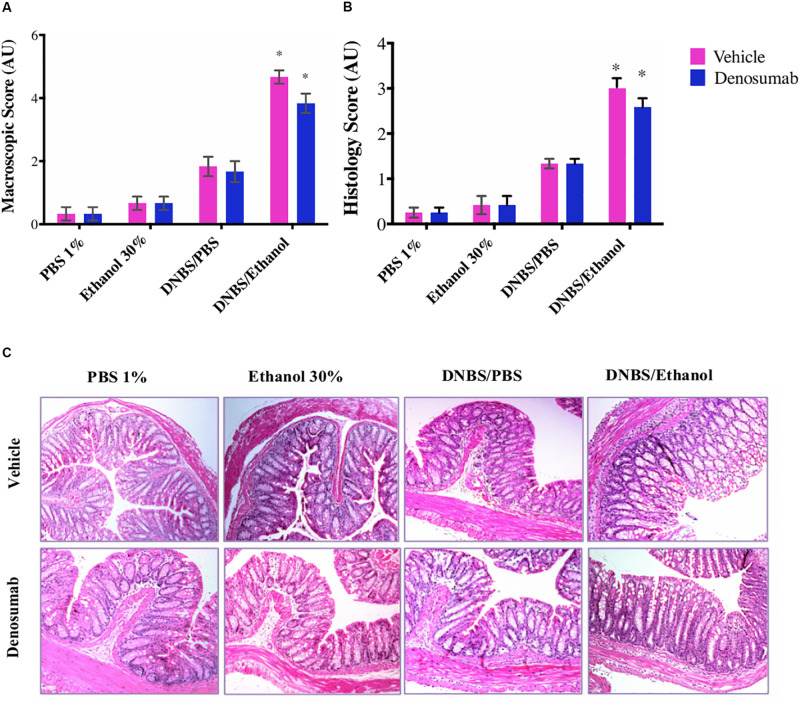
The effects of DNBS/Ethanol-induced colitis and denosumab treatment on macroscopic score and representative hematoxylin and eosin (H&E) stained colon mucosa, histologic and score. **(A)** Colonic macroscopic score. **(B)** Histological scores. **(C)** The H&E stained colon mucosa section showed a significant disruption of epithelial integrity, necrosis and transmural infiltration of immune cells. DNBS/Ethanol group increased (*p* = 0.03) the histological score while denosumab did not alleviate colitis. AU: arbitrary unit. Error bars are shown as SEM. *p < 0.001 when compared with other induction models within the denosumab or vehicle group calculated using SAS MIXED procedure and illustrated by Prism using two-way ANOVA. *n* = 6 mice per group.

PBS 1%, Ethanol 30% or DNBS/PBS groups did not show any effect on the macroscopic index as no differences were detected in rectal bleeding, rectal prolapse, diarrhea and colonic bleeding neither on the histological score (Figure 3A). However, compared to Ethanol treatment, DNBS/Ethanol increased the macroscopic score by 6-fold (*p* = 0.01, Figure 3A) and the histological score by 4-fold (Figures 3B,C). In non-colitic conditions (PBS 1%, Ethanol 30% or DNBS/PBS) and colitic conditions (DNBS/Ethanol), denosumab treatment did not modify either the macroscopic or the histological score (Figures 3A–C).

### Effect of Denosumab on Colonic and Serum Acute Inflammatory Markers

**FIGURE 4:**
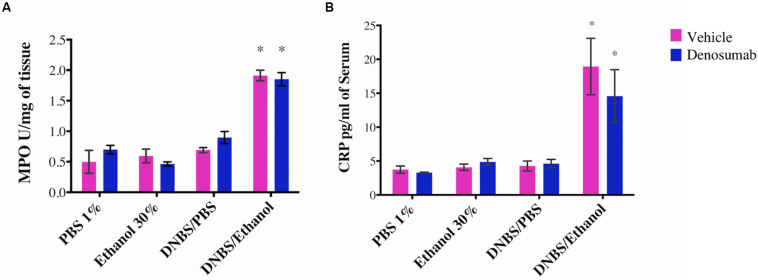
The effects of DNBS/Ethanol-induced colitis and denosumab treatment on regulating the colonic MPO activity and serum CRP. DNBS/Ethanol significantly increased **(A)**. Colonic MPO activity and **(B)**. Serum CRP. Denosumab did not ameliorate the negative effects of DNBS/Ethanol-induced colitic conditions. Vehicle = PBS 1%. Error bars are shown as SEM. *p < 0.001 when compared with other induction models within the denosumab or vehicle group, is calculated using SAS MIXED procedure and illustrated by Prism using two-way ANOVA. *n* = 6 mice per group.

PBS 1%, Ethanol 30% or DNBS/PBS groups did not show any effect on the colonic MPO activity and no differences were detected in the serum CRP level. Compared to ethanol treatment, DNBS/Ethanol increased the colonic MPO activity by 3.2-fold (*p* = 0.01, Figure 4A) and the serum CRP level by 4.74-fold (Figure 4B). In non-colitic conditions (PBS 1%, Ethanol 30% or DNBS/PBS) and colitic conditions (DNBS/Ethanol), denosumab treatment did not modify either the colonic MPO activity or serum CRP level (Figures 3A,B).

### Effect of Denosumab on Colonic Pro-inflammatory Cytokines

**FIGURE 5:**
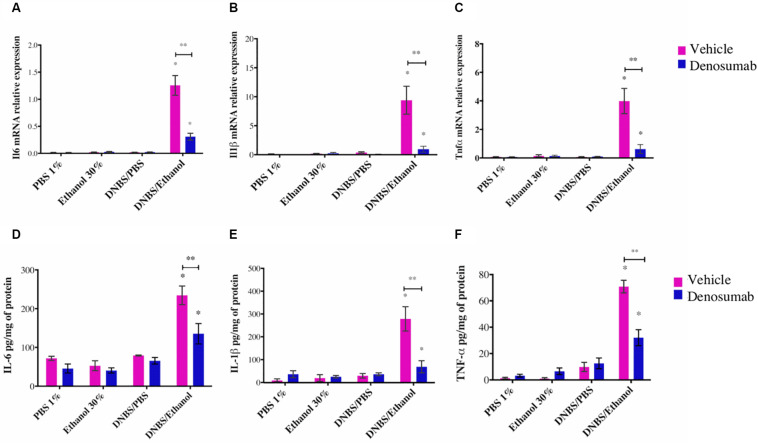
The effects of DNBS/Ethanol-induced colitis and denosumab treatment on the colonic IL1β, IL-6, and TNF-α pro-inflammatory cytokines. DNBS/Ethanol increased (*p* < 0.05) colonic pro-inflammatory cytokines **(A–C)**. The cytokine expression levels were confirmed using qRT-PCR analysis **(D–F)**. TATA Box Binding Protein (TBP) is used as housekeeping genes for qRT-PCR. Error bars are shown as SEM. ^∗^*p* < 0.001 when compared with other induction models within the denosumab or vehicle group, and ^∗∗^*p* < 0.001 when compared one induction model within the denosumab and vehicle group. calculated using SAS MIXED procedure and illustrated by Prism using two-way ANOVA. *n* = 6 mice per group.

PBS 1%, Ethanol 30% or DNBS/PBS groups did not show any effect on colonic mRNA expression of *Il1*β, *Il6* and *tnf*α and protein level (Figures 5A–F). Compared to Ethanol treatment, DNBS/Ethanol increased (*p* = 0.03) mRNA expression and protein level of *Il1*β by 10-and 5.5-fold, *Il6* by 5 and 1.5-fold, and *Tnfa* by 3 and 1.3-fold, respectively (Figures 5A–F). In colitic conditions (DNBS/Ethanol), denosumab treatment decreased significantly the mRNA and protein level of IL-1B, IL-6 and TNF-α (Figures 5A–F). In non-colitic conditions (PBS 1%, Ethanol 30% or DNBS/PBS) and colitic conditions (DNBS/Ethanol), denosumab treatment did not modify the markers studied (Figures 5A–F).

### Colonic and Fecal Alpha-Diversity

In Chao1 rarefaction plots, *X*-axis indicates the rarified 1,600 and 25,000 sequences per sample for colon and fecal samples, respectively, while *Y*-axis represents the Chao1-index of species richness. SAS MIXED procedure was used to calculate p-value and R software for plotting of the alpha diversity graphs (Supplementary Figures S1, S2). As the Chao1 rarefaction graphs show (Supplementary Figures S1A,B), both resident colon microbiota and the transient fecal microbiota showed a change in their composition. The data indicate that in the colon samples, within the vehicle group, all induction models increased the chao1-index compared to 1% PBS, while DNBS/PBS resulted in the most significant increase in the species richness (*p* = 0.021) (Supplementary Figure 2A). In the case of fecal samples (Supplementary Figure 2B), in the absence of denosumab, all induction models reduced (*p* = 0.03) chao1-index notably. In the DNBS/Ethanol colitic condition, there was a decrease (*p* = 0.03) from PBS 1% in the average chao1-index from 3,560 to 2,620 (Supplementary Figure 2A). After 4 days continuous i.p. injection of denosumab, chao1 index of α-diversity of all induction models stayed at a similar level (*p* = 0.2).

### Colonic and Fecal Beta-Diversity

**FIGURE 6:**
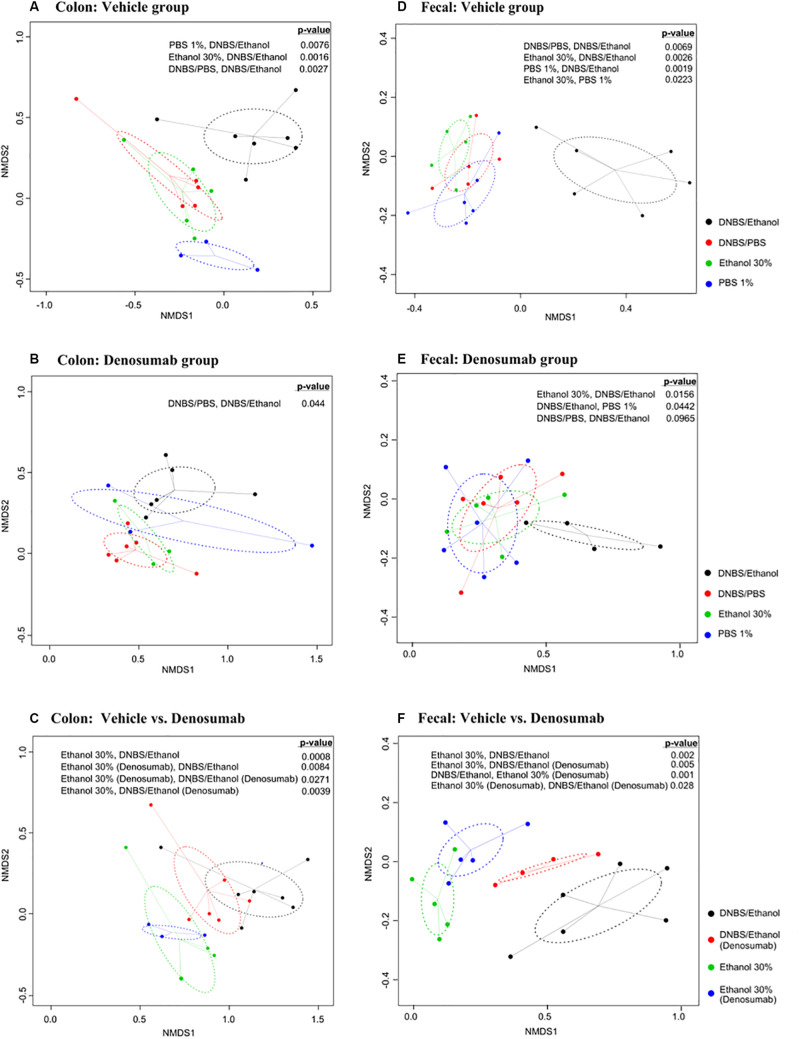
Non-metric multidimensional scaling (nMDS) ordination plot, a measure of relative dissimilarities in the bacterial community composition in the colon mucosa and feces of **(A,D)** vehicle and **(B,E)** denosumab-treated mice; **(C,F)** comparison between two groups of denosumab and vehicle in DNBS/Ethanol vs. Ethanol treatments. The colored points are shaded according to different treatment groups. The *p*-values were calculated using PERMANOVA. For the multiple-comparison tests, only the significant p-values were included. Trends were discussed at *p* < 0.1.

To compare diversity between different induction models within denosumab and vehicle groups, nMDS plots based on Bray-Curtis dissimilarity matrices were generated. Colonic mucosa-associated microbiota clustered separately (*p* = 0.002) in DNBS/Ethanol compared to other induction models in vehicle group. The same pattern was observed in fecal samples of vehicle group. Additionally, the fecal microbiota of 1% PBS and 30% Ethanol were clustered distinctly (*p* = 0.02) from each other. In the colonic mucosa, there was clear evidence of alleviating the dysbiotic effects of DNBS/Ethanol in denosumab-treated animals according to Bray-Curtis clustering patterns. This pattern was similarly observed in fecal samples (Figure 6) and denosumab could limit dysbiosis in the transient microbiota within 1% PBS and 30% Ethanol treated controls. Moreover, DNBS/Ethanol with denosumab-treatment has a trend toward the Ethanol 30% in both vehicle and denosumab groups (*p* = 0.1) with a significant difference in the beta-diversity between DNBS/Ethanol of vehicle group and both Ethanol 30% of vehicle and denosumab-treated groups (Figures 6C,F).

### Clustering of Colonic and Fecal of Microbiota

**FIGURE 7:**
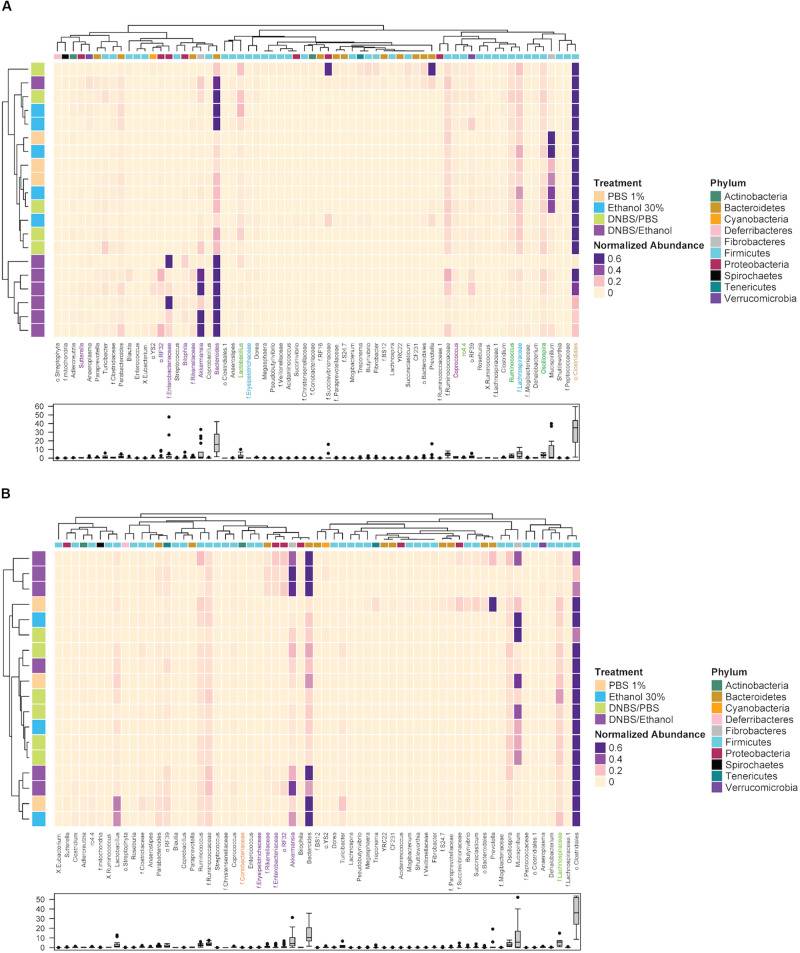
The clustering pattern of colonic and fecal microbial communities of vehicle and denosumab-treated samples. Rows correspond to samples and columns correspond to abundant genera (>0.1% of community). The “normalized abundance” key relates colors to the normalized proportions of genera (relative abundance of each genus divided by the Euclidean length of the column vector). The left dendogram shows how samples are clustered based on their Bray–Curtis dissimilarities (using unweighted pair group method with arithmetic averaging UPGMA). The significance of clustering patterns has been calculated based on 9999 permutations and p-values calculated based on PERMANOVA. The top dendogram shows how genera correlate (co-occur) with each other based on their Spearman’s correlation coefficient. The “Phylum” key relates the top annotations to the corresponding phylum of each genus. The “Treatment,” key relates samples to the treatments group (PBS 1%, Ethanol 30%, DNBS/PBS, DNBS/Ethanol). The bottom box-plot shows the distributions of the non-normalized relative abundances of genera in vehicle group **(A)** and denosumab group **(B)**. Color codes have been also used to highlight bacterial genera that were significantly associated with treatment groups (colors are in accordance with the colors of treatment groups; *p* < 0.05).

Next, a clustering analysis based on Bray-Curtis dissimilarity was employed in R (Gu et al., 2016) to investigate which phyla and genera, which were responsible for the gut dysbiosis and whether the clustering pattern of microbiota at the genus level changed following DNBS/Ethanol colitis induction and denosumab administration. We found that the vehicle group of both colon mucosa (*Y*-axis of Figure 7A), the DNBS/Ethanol induction model clustered separately from its control groups (*p* = 0.03), while DNBS/PBS, Ethanol 30%, and PBS 1% didn’t cluster separately. Bacteroidetes, Fibrobacteres, and Proteobacteria were the phyla with highly associated relative abundances with DNBS/Ethanol both in the colon mucosa of vehicle mice (*p* = 0.01). In contrast, phylum Firmicutes was highly associated with colon mucosa in the vehicle group (Figure 7A). The clustering analysis of denosumab-treated group showed a clear alteration in clustering pattern in both colon mucosa microbiota (Figure 7B). There was no significant difference between clustering of DNBS/Ethanol group vs. its controls.

Following up on the previous analyses, using LEfSe and MIXED procedure of SAS, the most significant changes in the relative abundance of bacterial taxa were identified. In the vehicle group, DNBS/Ethanol decreased Lachnospiraceae and Clostridia from Firmicutes phylum (*p* = 0.02) and increased Bacteroidaceae and *Akkermansia* from Bacteriodetes and Veruccomicrobia (*p* = 0.01), respectively. Denosumab treatment tended (*p* = 0.1) to increase the Lachnospiraceae and Clostridia compensating for their low abundance in the colon mucosa of DNBS/Ethanol and DNBS/PBS induction models. Denosumab treatment also decreased the relative abundance of Bacterioidaceae in the abovementioned groups (*p* = 0.02).

### Correlation Analysis

**FIGURE 8:**
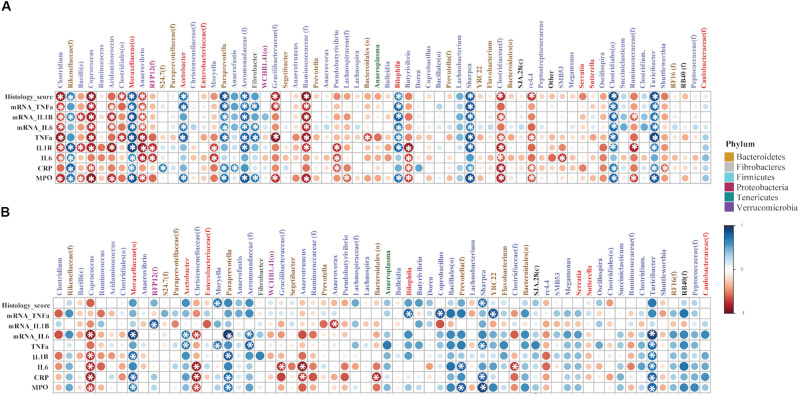
The correlation coefficient between the proportion of abundant colonic bacteria taxa (≥0.5% of community) and immunological factors (inflammatory markers), in both vehicle and denosumab-treated mice. The non-parametric Spearman’s rank correlation implemented in PAST software was used. For each correlation, correlation coefficient (Spearman’s Rho) and p-value were obtained and the resulting correlation matrix was visualized in a heatmap format generated by the corrplot package of R ver. 02-0.2010. The correlation coefficient values ranged from -1 (red) to +1 (blue) with larger absolute values indicating stronger relationship while positive and negative values between **(A)** DNBS/Ethanol vs. Ethanol 30% and immunological factors indicating the direction of association and **(B)** in the presence of denosumab. Alpha value for the correlation confidence intervals was set up as 0.05. *p < 0.05.

The non-parametric Spearman’s rank correlation analysis showed associations between several bacterial taxa with an abundance ≥0.5% of community in the colon mucosa and inflammatory markers (IL-1β, IL-6, TNF-α at both protein and gene level), histologic score, CRP, and MPO. According to Figure 8A, the i.r. administration of DNBS/Ethanol compared to its control Ethanol 30% resulted in a notable dysbiosis as shown by white asterisk, while some genera were associated with a negative or positive correlation (*p* ≤ 0.05). In the presence of denosumab (Figure 8B), less significant numbers of correlation were observed, demonstrating the effective role of denosumab in alleviating the dysbiotic effect induced by DNBS/Ethanol.

### Prediction of the Functional Capacity of Microbiota

**FIGURE 9:**
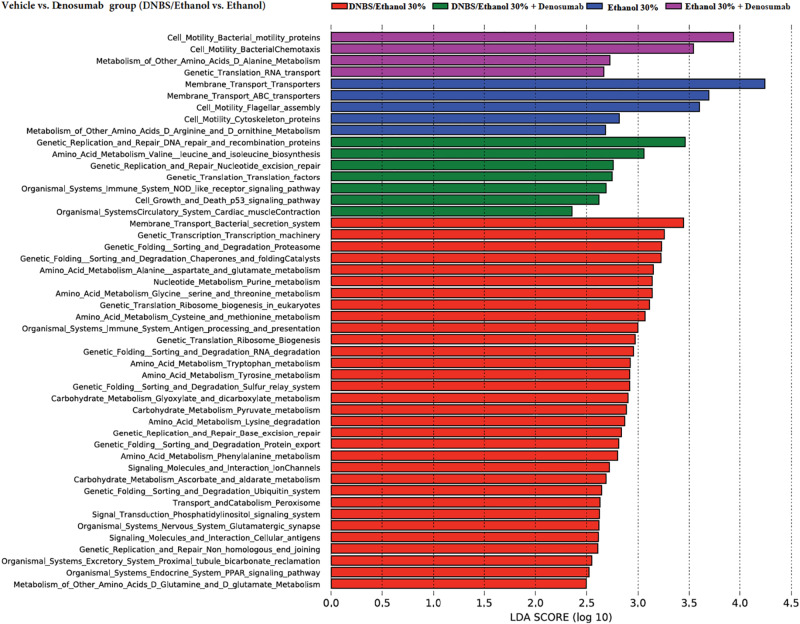
Prediction of functional capacity of colon-associated microbiota in vehicle vs. denosumab-treated mice. A statistical difference between KEGG pathways (explored at Levels 1–3, indicated as L1–L3) of predicted colonic mucosa metagenomes were evaluated by LEfSe, a metagenome analysis approach which performed the linear discriminant analysis following the Wilcoxon Mann–Whitney test to assess effect size of each differentially abundant variable. The length of the horizontal bars indicated log-fold changes for each variable. Color code represents the class of treatments; red and green represent colitic condition of DNBS/Ethanol, while blue and purple are showing the Ethanol 30% condition in the absence and presence of denosumab, respectively.

To assess the functional capacity of the microbiome under each treatment and induction model, PICRUSt was used. Several metabolic pathways were associated with DNBS/Ethanol-induced colitis in the vehicle group. Denosumab administration altered several KEEG pathways predicted by PICRUSt. Using LEfSe (Segata et al., 2011), several metabolic pathways that increased in association with each treatment were highlighted (Figure 9). Each color was assigned to one treatment. DNBS/Ethanol administration in the vehicle group increased several pathways, including amino acid uptake, carbohydrate synthesis, lipid uptake, and mucin overproduction metabolism while denosumab treatment reduced such pathways in DNBS/Ethanol colitis mice.

## Discussion

In this study, we investigated the role of the RANKL inhibitor, denosumab, on mucosal inflammatory markers, and the gut microbiota in DNBS-experimental colitis. Denosumab reduced the colonic expression of proinflammatory cytokines (IL-6, IL-1β TNF-α) and modulated the disruption of bacterial fecal and mucosal-associated microbiota. Therefore, this study suggests that denosumab is a potential future therapeutic strategy in the management of CD.

We found that denosumab treatment decreased significantly the expression of the proinflammatory cytokines (IL-6, IL-1β, TNF-α) at the colonic mucosa. The reduction in proinflammatory cytokines levels at the colonic mucosa can be interpreted as a RANKL blockade that led to the inhibition of the activation of immune cells expressing the RANK receptors (Wang et al., 2001; Ashcroft et al., 2003; Moelants et al., 2013). RANKL-Deficient mice are characterized by a reduced numbers of B-cells in the spleen and with defective transition of pro-B to pre-B resulting in an impaired antibody immune response in the invasion of pathogenic bacteria (Kong et al., 1999). Although denosumab reduced the colonic cytokines without a significant effect on the onset and the severity of colitis, this could be attributed to other immunoregulation and cell death processes (Eissa et al., 2019) that may override the effect of denosumab. Therefore, our study highlights the potential anti-inflammatory effect of RANKL inhibitor during the progression of colitis. One limitation of our experimental plan is the lack anti-inflammatory cytokine investigation; additional experiment targeting specific innate (anti-inflammatory macrophages) and adaptive (T-reg) immune cells need to be conducted.

Administration of DNBS alone or Ethanol alone either in the vehicle or denosumab group did not affect the colonic or fecal microbiota. Hence, the suggested mechanism behind DNBS/Ethanol role can be explained by the fact that Ethanol administration is needed to disrupt the colonic mucosal barrier and consequently let the DNBS to penetrate into lamina propria in order to haptenize the local colonic and gut bacterial proteins to acquire immunogenic characteristic (Morampudi et al., 2014). DNBS by its high-binding affinity to lysine e-amino group shifts those membrane-bound proteins to the haptenized proteins, thus, initiating the activation of antigen presenting cells inside the colon mucosa to overexpress pro-inflammatory cytokines, such as INF-γ, IL-β, IL-12, TNF-α and nitric oxide (NO) through induction of inducible nitric oxide synthase (iNOS) as well as T-helper 1-mediated innate immune response (Kim et al., 2012; Goyal et al., 2014; Morampudi et al., 2014).

Resident gut microbiota are key role players in IBD pathogenesis since approximately all IBD murine models require microbiome presence for development of colitis as germ-free mice show no signs for initiation of colitis (Rath et al., 2001). Several meta-analyses also have identified dysbiotic patterns of the fecal and the mucosa-associated microbiotas in both UC and CD patients and murine experimental models (Andoh et al., 2005; Moschen et al., 2005; Xenoulis et al., 2008; Zenewicz et al., 2008; Samanta et al., 2012; Knights et al., 2013; Wills et al., 2014; Halfvarson et al., 2017). There also is a significant decrease in Firmicutes and Clostridiales in IBD (Oberc and Coombes, 2015; Abeles et al., 2016). Interestingly, our study demonstrated that denosumab decreased the altered species richness and avoided dysbiosis in DNBS/Ethanol treatment and attenuated microbiota dysbiosis within both colon mucosa and feces. Denosumab treatment increased the level of f. Lachnospiraceae from p. Firmicutes in contrast with decreased level of o. RF32, f. Entereobacteriaceae, and g. *Bilophila* from p. Proteobacteria, g. *Bacteroides*, g. *Rikenellaceae* from p. Bacteroidetes, and g. *Akkermansia* from p. Verrucomicrobia.

Denosumab also increased o. Clostridiales. The role of g. *Clostridium* is widely investigated in butyrate production, a short-chain fatty acid, and source of energy for the intestinal epithelium with the potentiality to acidify the intestinal lumen and therefore protect it against certain pathogenic bacteria, such as *Salmonella* and *Escherichia coli* (Topping and Clifton, 2001; Vogt et al., 2015). Schwab et al. (2014) reported several genera from p. Bacteroidetes, such as g. *Bacteroides* as key players in the onset of a mouse model of colitis. Similarly, several clinical studies reported these bacteria in both CD and UC patients (Ott et al., 2004; Andoh et al., 2005; Swidsinski et al., 2005; Gophna et al., 2006; Sepehri et al., 2007; Bibiloni et al., 2008). Furthermore, we found that a significant decrease in species richness under DNBS/Ethanol and it was clustered far from its controls. This distinctive clustering pattern was also seen in colon mucosa, although, DNBS/Ethanol administration increased the species richness in colon mucosa compared to controls. Alpha-diversity data also confirmed the existence of dysbiosis within both resident and transient microbiota in the vehicle group, and the alleviating effect of denosumab in these samples.

The mechanism behind how these microbiota shifts contribute to pathophysiological state during CD and what initiates such dysbalanced microbiota in parallel with excessive inflammatory response is still an active area of research. One idea is the loss of SCFA producing bacteria lead to less enterocyte survival at epithelial layer and increase the permeability of this layer through loosen tight junction because of impaired butyrate synthesis and then as a result activate inflammatory response pathways (Peng et al., 2009). Several studies demonstrated the decreased abundance in the majority of Firmicutes members, such as g. *Clostridium* in CD patients with an increased abundance in the p. Proteobacteria (Manichanh et al., 2006; Oberc and Coombes, 2015). Also, survival of facultative anaerobic Proteobacteria, such as members of Entereobacteriaceae, and Bilophila with their higher robustness to reactive-oxygen species gives them priority to compete during inflammatory response with predominant anaerobic Firmicutes and Bacteroidetes (Miyoshi and Chang, 2017). Therefore, from CD studies it seems that microbiome dysbiosis works as both as a cause and a consequence of CD pathogenesis and it appears that this conclusion can be extended to the DNBS/Ethanol model as well.

## Conclusion

In conclusion, although no effect was visible on macroscopic markers and CRP, this study shows that denosumab, RANKL inhibitor, reduced the proinflammatory cascades in the colonic mucosa and also had a beneficial effect on the composition of gut microbiota during progression of DNBS colitis. These data suggest potential therapeutic strategies toward CD management.

## Data Availability Statement

The sequencing data are uploaded into the Sequence Read Archive (SRA) of NCBI (http://www.ncbi.nlm.nih.gov/sra) and can be accessed through accession numbers SRR1158862–SRR11588957.

## Ethics Statement

The animal study was reviewed and approved by University of Manitoba Animal Ethics Committee and conducted under the guidelines of the Canadian Council on Animal Care.

## Author Contributions

AK, NE, CB, and J-EG conceived and designed the experiments. AK, NE, PM, MR, KK, and LK performed the experiments. AK, NE, EK, and J-EG analyzed the data. AK, NE, and MR performed the research. EK and J-EG contributed to reagents, materials, and analysis tools. AK, NE, EK, CB, and J-EG wrote the manuscript. All authors have read and approved the manuscript.

## Conflict of Interest

CB has been on the advisory boards for Abbvie Canada, Janssen Canada, Shire Canada, Takeda Canada, Pfizer Canada, and consulted to Mylan Pharmaceuticals. He has received educational grants from Abbvie Canada, Pfizer Canada, Shire Canada, Takeda Canada, Janssen Canada and has been on the speaker’s panel for Janssen Canada, Takeda Canada, Shire Canada, and Medtronic Canada. The remaining authors declare that the research was conducted in the absence of any commercial or financial relationships that could be construed as a potential conflict of interest.

## Footnotes

1 http://blast.ncbi.nlm.nih.gov/Blast.cgi

2 http://www.ncbi.nlm.nih.gov/sra

3 https://cran.r-project.org/web/packages/corrplot/vignettes/corrplot-intro.html
