# Supplementary material for: Denosumab Regulates Gut Microbiota Composition and Cytokines in Dinitrobenzene Sulfonic Acid (DNBS)-Experimental Colitis
Source: Front Microbiol. 2020 Jun 25;11:1405. doi: 10.3389/fmicb.2020.01405 (PMC7331113; doi:10.3389/fmicb.2020.01405)
Supplement: Supplementary file 1 [file Data_Sheet_1.PDF]

Supplementary Information:

Denosumab regulates gut microbiota composition and cytokines in dinitrobenzenesulfonic acid (DNBS)-experimental colitis

Azin Khafipour[1], Nour Eissa[1, 2, 3, 5], Peris M. Munyaka[1, 3, 4], Mohammad F. Rabbi[1, 2, 3, 5], Kunal Kapoor[1, 3, 5], Laetitia Kermarrec[1, 3, 5], Ehsan Khafipour[4], Charles N. Bernstein[3, 5], Jean-Eric Ghia[1, 2, 3, 5*]

[1]Department of Immunology, University of Manitoba, Winnipeg, MB, Canada

[2]Children's Hospital Research Institute of Manitoba, University of Manitoba, Winnipeg, MB, Canada

[3]Section of Gastroenterology, Department of Internal Medicine, Rady Faculty of Health Sciences, University of Manitoba, Winnipeg, MB, Canada

[4] Department of Animal Science, University of Manitoba, Winnipeg, Manitoba, Canada

[5] University of Manitoba IBD Clinical and Research Centre, University of Manitoba, Winnipeg, MB, Canada

Corresponding address:

Dr. Jean-Eric Ghia, Jean-Eric.Ghia@umanitoba.ca or jeghia@yahoo.fr

(A) (B)

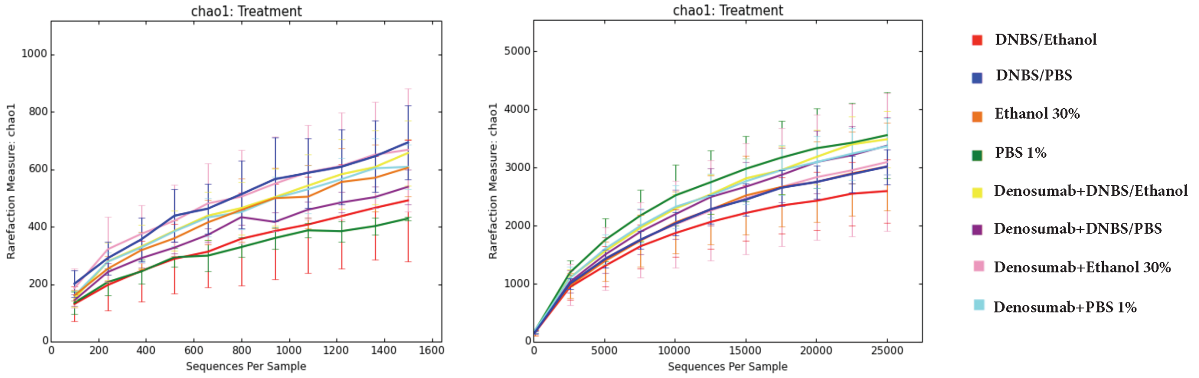

Supplementary Fig 1. Alpha-diversity of colonic and fecal microbiota of DNBS/Ethanol-induced colitis and denosumab-treated mice. Measure of Chao1 index of species richness based on operational taxonomic unit (OTU) in vehicle (i.r. administration of PBS 1%) and denosumab groups interactions, within colonic (A) and fecal (B) samples.

(A)

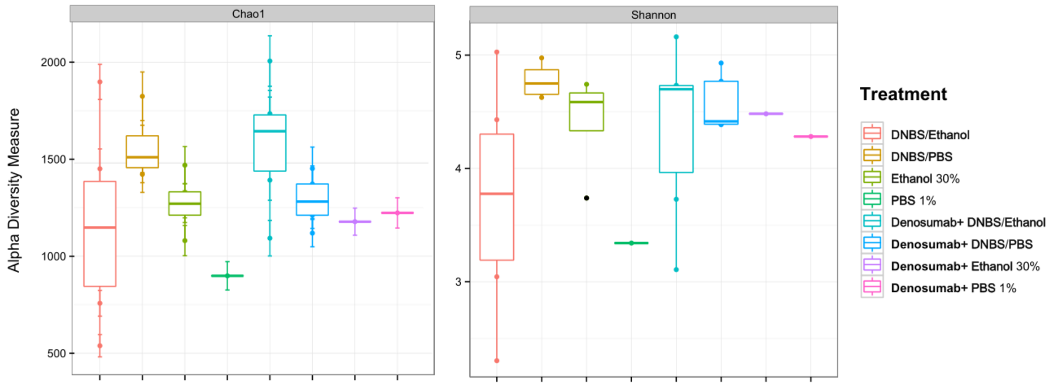

(B)

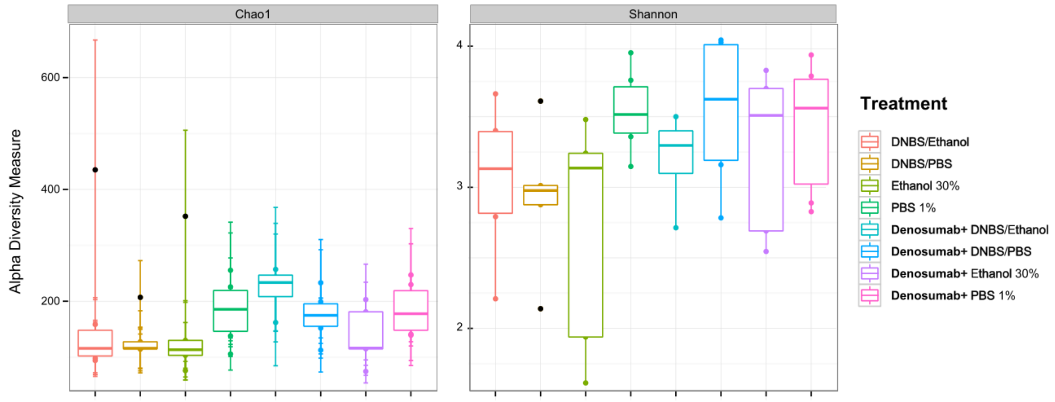

Supplementary Fig 2. Summary of alpha-diversity indices of vehicle- and denosumab-treated colon and vehicle samples. (A-B) Comparison of Chao1 and Shannon indices of colonic microbiota between denosumab-treated vs. vehicle animals with or without induction of DNBS/Ethanol colitis. Statistical analyses were conducted using MIXED procedure of SAS.
